# How to perform cost‐benefit analysis for interventions related to dementia

**DOI:** 10.1002/alz70860_104121

**Published:** 2025-12-23

**Authors:** Tiet‐Hanh Dao‐Tran, Tracy Comans, Namal BALASOORIYA, Digby SIMPSON, Lee‐Fay Low, Annica Barcenilla‐Wong, Paola VASQUEZ, Kim‐Huong Nguyen

**Affiliations:** ^1^ University of Queensland, Brisbane, QLD, Australia; ^2^ University of Queensland, Herston, QLD, Australia; ^3^ Griffith University, Gold Coast, QLD, Australia; ^4^ The University of Sydney, Sydney, NSW, Australia; ^5^ University of Sydney, Licombe, NSW, Australia

## Abstract

**Background:**

Cost‐benefit analysis has recently been increasingly used to evaluate the value of interventions for dementia. This study aims to synthesise the data analysis approach, cost and benefit items, data sources, and valuation methods used in cost benefit analyses of interventions for dementia.

**Method:**

A scoping review was conducted to address the research aims. Comprehensive systematic searches were performed on in PubMed, MEDLINE, CINALH, Embase, Scopus, PsycINFO and Econlit electronic databases for peer‐reviewed original articles published in English from January 2010 to December 2023 (i) performed a CBA of interventions for dementia (ii) described either cost or benefit items; (iii) performed quantitative data analysis on either costs or benefits. Two authors independently screened and extracted data. The PRISMA Extension for Scoping Reviews Checklist was used to guide the report writing.

**Result:**

The review included 15 studies. Both traditional CBA approach and its integration with the social return on investment (SROI) approach have been used for CBA of interventions for dementia. The set of cost and benefit items may vary depending on the intervention. Staff training, intervention supplies, building hire, and transportation were common cost items. Quality‐adjusted life years (QALY), general practitioner visits, and emergency room visits were common benefit items. Cost data were often sourced from the study budget/ assumptions. Market and shadow pricing were used for cost valuation. Benefit data were often sourced from the social value banks and literature. The value of statistical life was frequently used for benefit valuation.

**Conclusion:**

Methodology approaches for CBA of interventions for dementia are heterogeneous. The findings provide helpful information for considering methodological approaches in future CBA of interventions for dementia and similar interventions or conditions.

